# Supplementary material for: A single-cell transcriptomic atlas of the developing chicken limb
Source: BMC Genomics. 2019 May 22;20:401. doi: 10.1186/s12864-019-5802-2 (PMC6530069; doi:10.1186/s12864-019-5802-2)
Supplement: Supplementary file 1 — Figure S1. Sample compositions and data statistics. (a) Cellular composition of the samples and datasets, color code corresponds to Fig. 2a-c. (b) UMI count distributions across the samples. (c) Gene count distributions across the samples. Figure S2. Expression patterns of marker genes. Related to Fig. 1. Normalized expression patterns of selected genes to identify the different cell populations in our broad clustering, plotted on the tSNEs from sample (a) HH25, (b) HH29 and (c) HH31. Figure S3. Co-expression modules expression patterns. Related to Fig. 3. Average expression of each WGCNA co-expression module on the tSNE of sample HH29. (PDF 4613 kb) [file 12864_2019_5802_MOESM1_ESM.pdf]

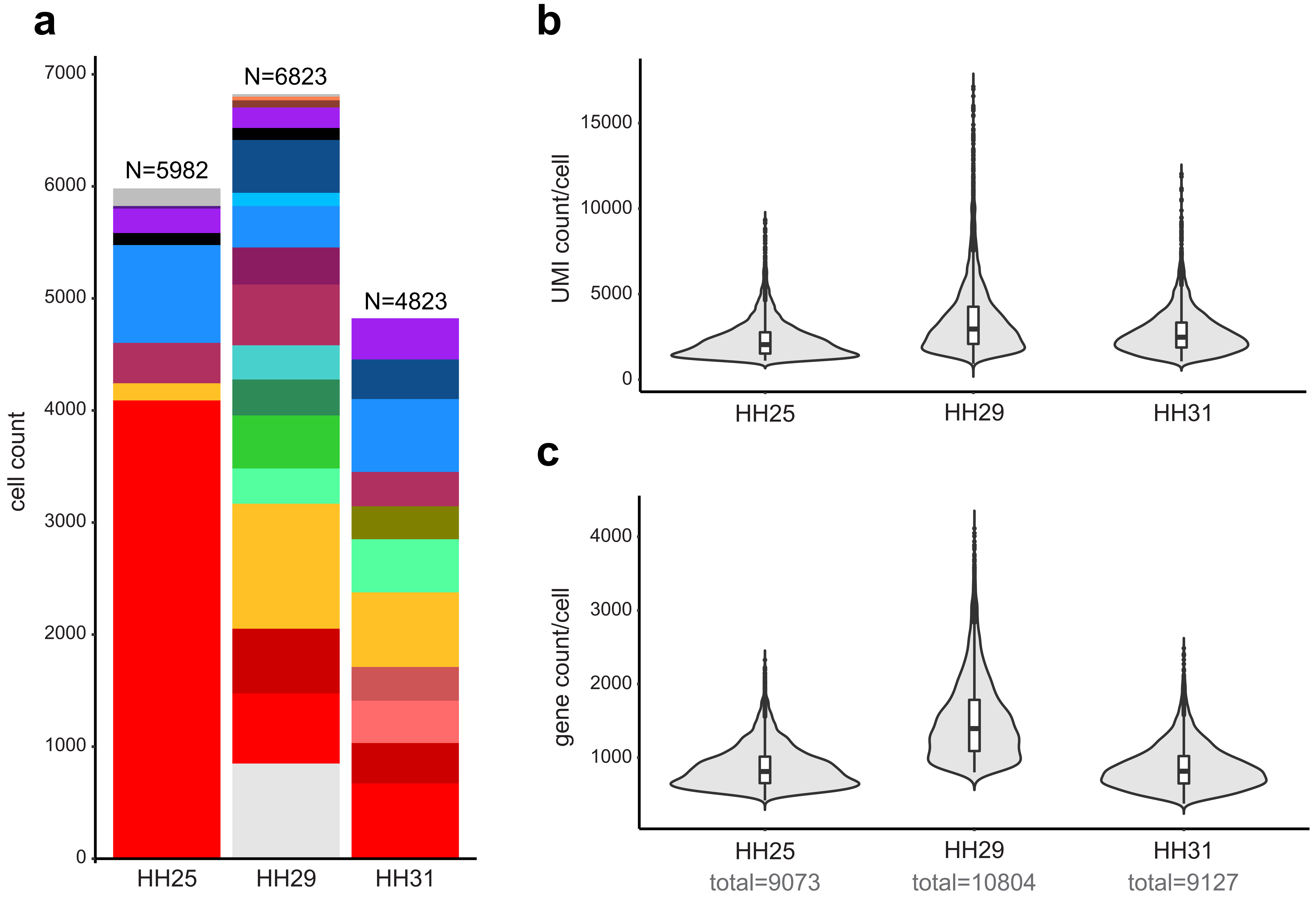

Fig. S1
Sample compositions and data statistics. (a) Cellular composition of the samples and datasets, color code corresponds to Fig. 2a-c. **(b)** UMI count distributions across the samples. **(c)** Gene count distributions across the samples.

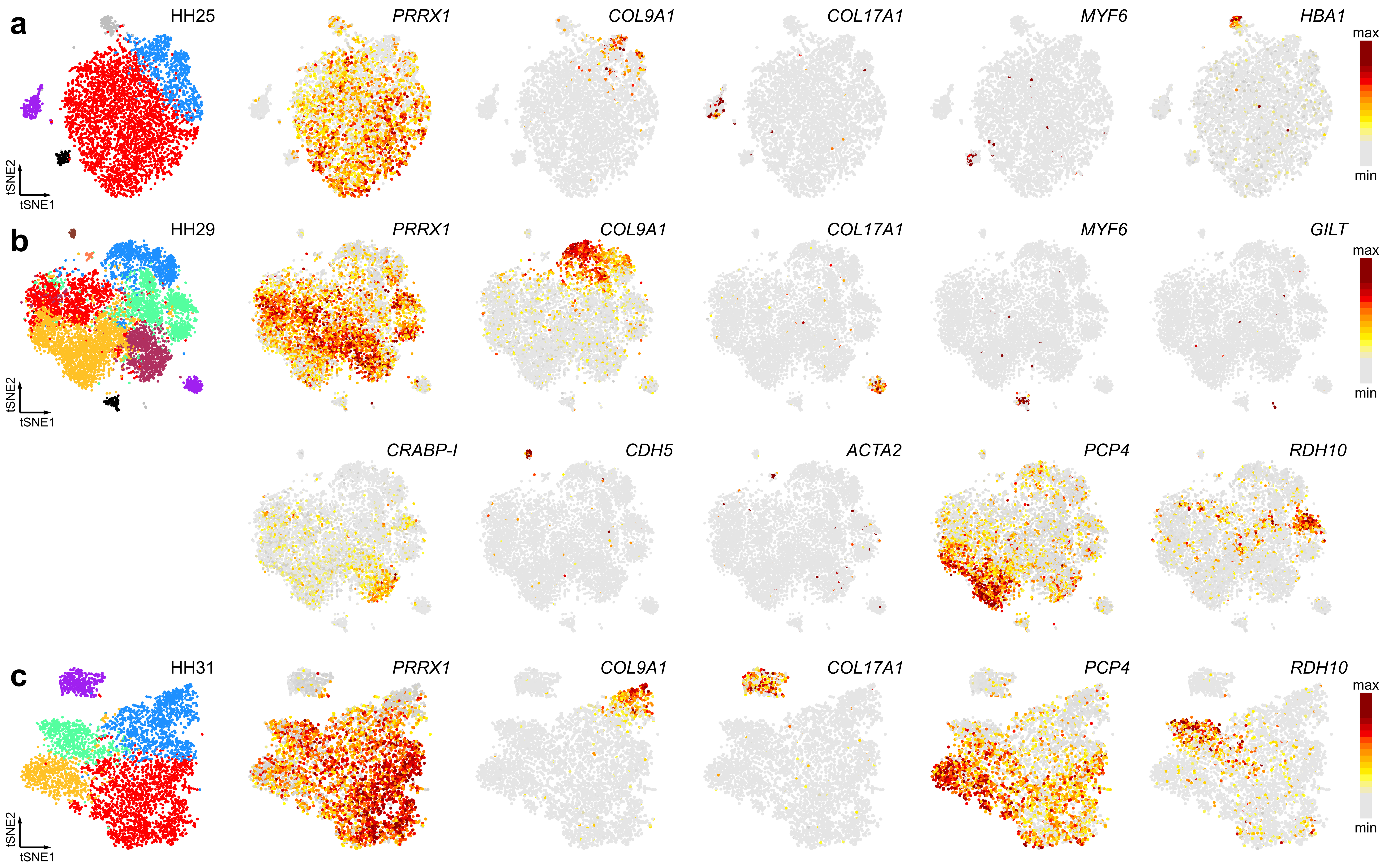

Fig. S2

Expression patterns of marker genes. Related to Fig. 1. Normalized expression patterns of selected genes to identify the different cell populations in our broad clustering, plotted on the tSNEs from sample **(a)** HH25, **(b)** HH29 and **(c)** HH31.

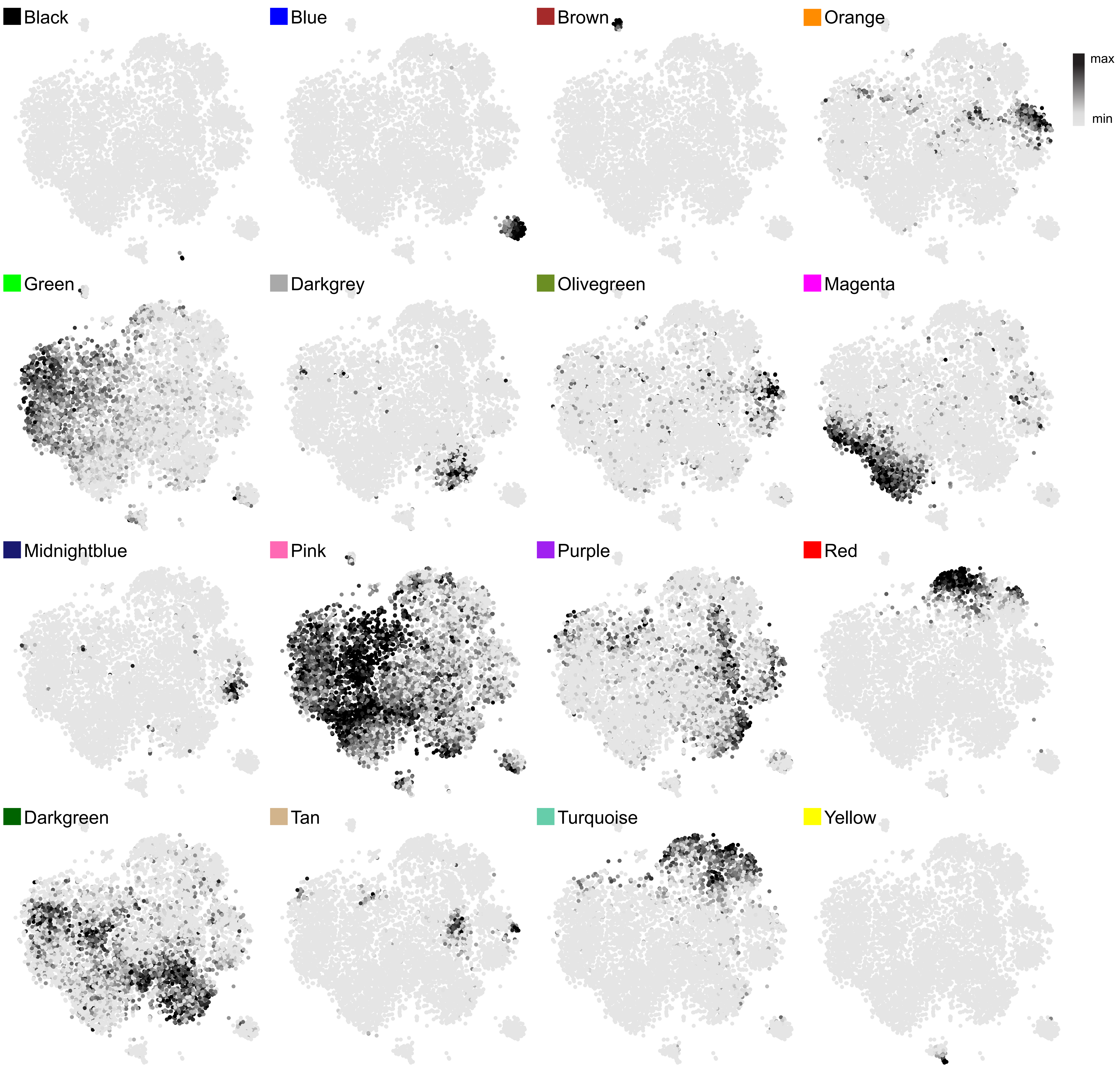

Fig. S3

Co-expression modules expression patterns. Related to Fig. 3. Average expression of each WGCNA co-expression module on the tSNE of sample HH29.
